# Association between peripheral retinal lesions and ocular biometric parameters in adults with mild-to-moderate myopia attending a refractive surgery clinic

**DOI:** 10.3389/fmed.2026.1929247

**Published:** 2026-09-18

**Authors:** Zujian Zhu, Benqian Liu, Zhentao Zhu, Shandi Liu, Ligang Jiang

**Affiliations:** 1 Jiangsu Health Vocational College, Nanjing, Jiangsu, China; 2 Lianyungang Eye Hospital, Lianyungang, Jiangsu, China; 3 Huai'an Clinical Medical College of Jiangsu University, Huaian Hospital of Huaian City, Huaian, Jiangsu, China; 4 Department of Ophthalmology, Quzhou People’s Hospital, The Quzhou Affiliated Hospital, Wenzhou Medical University, Quzhou, Zhejiang, China

**Keywords:** axial length, corneal curvature, mild-to-moderate myopia, peripheral retinal lesions, spherical equivalent, white without pressure

## Abstract

**Background:**

To investigate the association between peripheral retinal lesions (PRLs) and ocular biometric parameters in adults with mild-to-moderate myopia.

**Methods:**

This retrospective cross-sectional study included 106 adults who contributed 171 eligible eyes with mild-to-moderate myopia. Based on dilated fundus examination and ultra-widefield imaging, 70 eyes were assigned to the PRL group and 101 eyes to the normal-fundus (NF) group. Participant age and eye-level spherical equivalent (SE), axial length (AL), mean corneal power (Km), corneal curvature radius (CR), and the exploratory corneal-curvature-adjusted parameter SEAL were compared. Eye-level continuous comparisons used participant-clustered robust standard errors. Lesion types and quadrant distributions were recorded descriptively.

**Results:**

Mean age did not differ between the groups (21.17 ± 3.76 vs. 22.04 ± 3.13 years, *P* = 0.200). SE was also similar (−4.23 ± 1.26 vs. −4.20 ± 1.07 D, *P* = 0.913). Compared with NF eyes, PRL eyes had a longer AL (26.39 ± 0.87 vs. 24.78 ± 0.72 mm, *P* < 0.001), lower Km (41.40 ± 0.90 vs. 44.51 ± 0.72 D, *P* < 0.001), higher CR (8.16 ± 0.18 vs. 7.59 ± 0.12 mm, *P* < 0.001), and a more negative SEAL value (−4.59 ± 1.42 vs. −3.95 ± 1.05 D, *P* = 0.009). White without pressure, lattice degeneration, and snowflake degeneration were the most frequent lesions. Quadrant involvement was most common superiorly, superotemporally, inferiorly, and inferotemporally.

**Conclusion:**

In this refractive-surgery-clinic sample, PRLs were associated at the eye level with longer AL, flatter corneas, and more negative exploratory SEAL values. Because the study was cross-sectional and did not include multivariable modeling, these findings do not establish independent predictors or causal relationships.

## 1 Introduction

Myopia is a highly prevalent refractive error in which parallel light rays focus anterior to the retina when accommodation is relaxed, resulting in blurred distance vision (1, 2). Its prevalence has risen rapidly worldwide, particularly in East Asia, where more than 80% of adolescents in some settings are affected (3, 4). A substantial proportion progress to high myopia, conventionally defined as SE ≤ −6.00 D (5). Axial length is an important structural indicator of myopia progression, particularly in East Asian populations (6). Contemporary myopia-control and refractive-surgery studies increasingly assess SE together with AL, corneal curvature, and other ocular geometric variables rather than relying on refraction alone (7–11). The World Health Organization recognizes uncorrected refractive error and myopia-related complications as major contributors to visual impairment, making myopia prevention and control an important public-health challenge (12–14).

The consequences of myopia extend beyond blurred distance vision. Myopia, particularly high myopia, is associated with several potentially irreversible ocular disorders (15). A shift of approximately 6.00 D toward greater myopia has been associated with a markedly increased risk of retinal detachment (16). Population-based evidence also suggests that myopia-related retinal microstructural patterns are associated with retinal-detachment risk (17). Increasing AL and myopia severity are additionally associated with myopic macular degeneration (18), open-angle glaucoma (19), and structural changes involving the optic nerve head, retinal nerve-fiber layer, and ganglion-cell complex (20). These complications can impair visual function and quality of life.

Peripheral retinal lesions (PRLs) are clinically important findings in myopic eyes and include peripheral retinal degenerations as well as related retinal breaks (21–24). They are frequently asymptomatic and may be detected during preoperative screening. Some lesion types, particularly those associated with vitreoretinal traction or retinal breaks, may increase the risk of rhegmatogenous retinal detachment (25).

The prespecified PRL categories in this study were white without pressure (26), snowflake degeneration (27), lattice degeneration (28, 29), peripheral cystoid degeneration (30), snail-track degeneration (31), pigmentary degeneration (32), and retinal tear (33). Retinal tear is a retinal break rather than a degeneration; therefore, PRL is used as the umbrella term throughout this manuscript. These entities have distinct morphological features and clinical implications, and lattice degeneration is particularly associated with retinal holes and tractional tears (25).

Early identification of PRLs in adults with mild-to-moderate myopia may improve preoperative peripheral retinal assessment. This study therefore aimed to describe the spectrum and distribution of PRLs and to examine their unadjusted associations with ocular biometric parameters in patients attending a refractive surgery clinic.

## 2 Subjects and methods

### 2.1 Subjects

This retrospective cross-sectional study screened the records of adults with mild-to-moderate myopia who attended the Refractive Surgery Clinic of Lianyungang Eye Hospital between June and August 2025. The sampling frame consisted of clinic patients seen during this period. Records were screened according to the prespecified eligibility criteria before classification by peripheral retinal status. When both eyes met the eye-level eligibility requirements, both were included. The final analytic sample comprised 106 participants contributing 171 eligible eyes. Of these, 70 eyes with at least one prespecified lesion formed the PRL group, and 101 eyes without a significant peripheral retinal abnormality formed the NF group.

Inclusion criteria were: (1) age ≥ 18 years; (2) mild-to-moderate myopia, operationally defined as −6.00 D < SE ≤ −0.50 D; and (3) no systemic disease or inherited ocular disorder likely to affect the retina. Exclusion criteria were: (1) previous refractive or other ocular surgery; (2) a history of retinopathy of prematurity, retinal detachment, or retinitis pigmentosa; (3) a comprehensive retinal examination prompted by acute ocular symptoms; and (4) corneal scar, cataract, or other media opacity that precluded reliable examination or imaging.

All participants underwent standardized examinations. The study adhered to the Declaration of Helsinki and was approved by the Ethics Committee of Lianyungang Eye Hospital (approval No. 2026005).

### 2.2 Methods

All participants underwent a comprehensive ophthalmic examination, including anterior-segment evaluation with a TOPCON SL-D7 slit lamp, refractive-error measurement with a NIDEK AR-1 autorefractor, AL measurement with the IOLMaster 700, corneal topography and curvature assessment with the OCULUS Pentacam, and ultra-widefield fundus imaging with the Optos system.

After pharmacologic mydriasis, two retinal specialists independently examined each eligible eye by slit-lamp biomicroscopy with a Volk 90D lens and independently reviewed the ultra-widefield images. Ultra-widefield imaging was used to document and localize lesions but did not replace the dilated clinical examination.

Operational definitions were prespecified as follows. White without pressure was a flat, translucent gray-white peripheral retinal area with a sharply demarcated or scalloped border visible without scleral indentation. Snowflake degeneration consisted of small yellow-white reflective or crystalline dots between the ora serrata and equator. Lattice degeneration was a sharply demarcated circumferential or linear area of retinal thinning, with or without pigmentation, branching white lines, atrophic holes, or marginal vitreoretinal traction. Peripheral cystoid degeneration consisted of clusters of tiny yellow-gray vesicular changes in the far peripheral retina (26).

Snail-track degeneration was an elongated, glistening white band of closely packed white dots with underlying retinal thinning. Pigmentary degeneration was recorded as localized peripheral retinal pigment epithelial clumping or reticular pigmentary change. A retinal tear was defined as a full-thickness neurosensory retinal break, including flap (horseshoe) and operculated tears (33). Disagreements were adjudicated by a third senior retinal specialist. Interobserver agreement was not quantified, and masking to biometric measurements was not documented.

The primary comparisons were participant age and eye-level SE, AL, Km, CR, and SEAL between the PRL and NF groups. The types of PRLs and their distribution across fundus quadrants were also recorded. Because retinal tears were included, the broader term PRL was used for the study outcome.

In addition to conventional ocular parameters, this study retained SEAL as an exploratory, study-specific corneal-curvature-adjusted transformation of SE. Its purpose was to rescale SE according to corneal geometry rather than to measure AL directly. SEAL was calculated using the following formula:

$$SE_{\mathrm{AL}}=\frac{5.5\times \mathrm{SE}\times \mathrm{CR}}{K_{m}}$$

Here, SE denotes spherical equivalent, CR denotes corneal curvature radius, and Km denotes mean corneal power. The coefficient 5.5 approximates the ratio of reference Km (approximately 43 D) to reference CR (approximately 7.85 mm), based on values reported in previous cohorts (34, 35). At these reference values, SEAL is approximately equal to SE.

For example, with SE = −4.00 D, SEAL equals −4.76 D when Km = 40 D and CR = 8.65 mm, approximately −4.02 D when Km = 43 D and CR = 7.85 mm, and −3.51 D when Km = 46 D and CR = 7.34 mm. Thus, for the same SE, a flatter cornea produces a more negative SEAL value, whereas a steeper cornea produces a less negative value. Because CR is derived from Km (CR = 337.5/Km) and the formula contains no AL term, SEAL was analyzed only as an exploratory transformation and not as a validated measure of AL-related risk.

Statistical analyses were performed using SPSS version 22.0. Normality was assessed using the Shapiro–Wilk test, and continuous data are presented as mean ± standard deviation. Participant-level age was compared using Welch's independent-samples *t* test. Eye-level SE, AL, Km, CR, and SEAL were compared using unadjusted linear models with participant-clustered robust standard errors to account for inclusion of both eyes from some participants. For an exploratory categorical analysis, eyes were stratified as long AL (>26.0 mm) or medium-to-short AL (≤26.0 mm), and PRL proportions were compared using Pearson's *χ*² test. No multivariable logistic-regression model was fitted; consequently, all reported associations are unadjusted. A two-sided *P* value < 0.05 was considered statistically significant.

## 3 Results

### 3.1 Comparison of participant and eye characteristics between the PRL and NF groups

The analytic dataset comprised 106 participants contributing 171 eyes: 70 PRL eyes and 101 NF eyes. Participant-level mean age was 21.17 ± 3.76 years for the PRL group and 22.04 ± 3.13 years for the NF group (*P* = 0.200). Eye-level SE values were similar (*P* = 0.913), whereas AL was longer in the PRL group (*P* < 0.001). Detailed comparisons are presented in Table 1.

**Table 1:** Comparison of general clinical characteristics between the PRD $\bar{x}\;\;\pm s$ group and the NF group $\left(\bar{x}\;\;\pm s\right)$.

Group	Number of cases (number of eyes)	Gender (Example)		Age (years)	SE (D)	AL (mm)
		Man	Woman			
PRD group	70 (70)	32	38	21.17 ± 3.76	−4.26 ± 1.13	25.69 ± 0.83
NF group	101 (101)	49	52	22.04 ± 3.13	−4.20 ± 1.07	24.78 ± 0.72
*t*/*x*^2^	–	0.129	−1.291	−0.158	10.268	–
*P*	–	0.719	0.200	0.964	<0.001	–

The PRD group refers to the group with peripheral retinal degeneration; the NF group refers to the group with normal fundus.

### 3.2 Comparison of corneal curvature-related parameters between the PRL and NF groups

Km was lower and CR was higher in PRL eyes than in NF eyes (*both P* < 0.001). The exploratory SEAL value was more negative in the PRL group (−4.59 ± 1.42 vs. −3.95 ± 1.05 D; *P* = 0.009). Because CR is mathematically derived from Km and SEAL incorporates both variables, these findings should not be interpreted as independent effects. Detailed data are presented in Table 2.

**Table 2:** Comparison of Km, CR, and SEAL values between patients in the PRD $\bar{x}\;\;\pm s$ group and the NF group $\left(\bar{x}\;\;\pm s\right)$.

Group	Number of eyes	Km (D)	CR (mm)	SEAL (D)
PRD group	70	41.40 ± 0.90	8.16 ± 0.18	−4.59 ± 1.42
NF group	101	44.51 ± 0.72	7.59 ± 0.12	−3.95 ± 1.05
*t*	–	−25.052	23.271	−3.222
*P*	–	<0.001	<0.001	0.002

The PRD group refers to the group with peripheral retinal degeneration; the NF group refers to the group with normal fundus.

### 3.3 Comparison of PRL proportions between axial-length categories

Among 47 eyes with AL > 26.0 mm, 45 (95.74%) had a PRL and 2 (4.26%) had a normal fundus. Among 124 eyes with AL ≤ 26.0 mm, 25 (20.16%) had a PRL and 99 (79.84%) had a normal fundus. The unadjusted PRL proportion differed between the two AL categories (*P* < 0.001). This exploratory cross-tabulation is presented in Table 3.

**Table 3:** Comparison of PRD incidence rates between patients with long axial length groups and those with medium-to-short axial length groups [eyes (%)].

Group	Number of eyes	PRD	Normal fundus
Long eye axis group	47	45 (95.74)	2 (4.26)
Short-to-medium axial length group	124	25 (20.16)	99 (79.84)
*x* ^2^	–	75.324	
*P*	–	<0.001	

### 3.4 Distribution of PRL types

Among the 70 PRL eyes, 12 eyes had two lesion categories. The 82 recorded lesion observations comprised white without pressure (33 eyes, 47.14% of PRL eyes), lattice degeneration (13 eyes, 18.57%), snowflake degeneration (12 eyes, 17.14%), retinal tear (8 eyes, 11.43%), peripheral cystoid degeneration (6 eyes, 8.57%), snail-track degeneration (5 eyes, 7.14%), and pigmentary degeneration (5 eyes, 7.14%). Because lesion categories were not mutually exclusive, counts sum to 82 and percentages sum to more than 100%. Representative appearances and detailed counts are shown in Figure 1 and Table 4, respectively.

**Figure 1:**
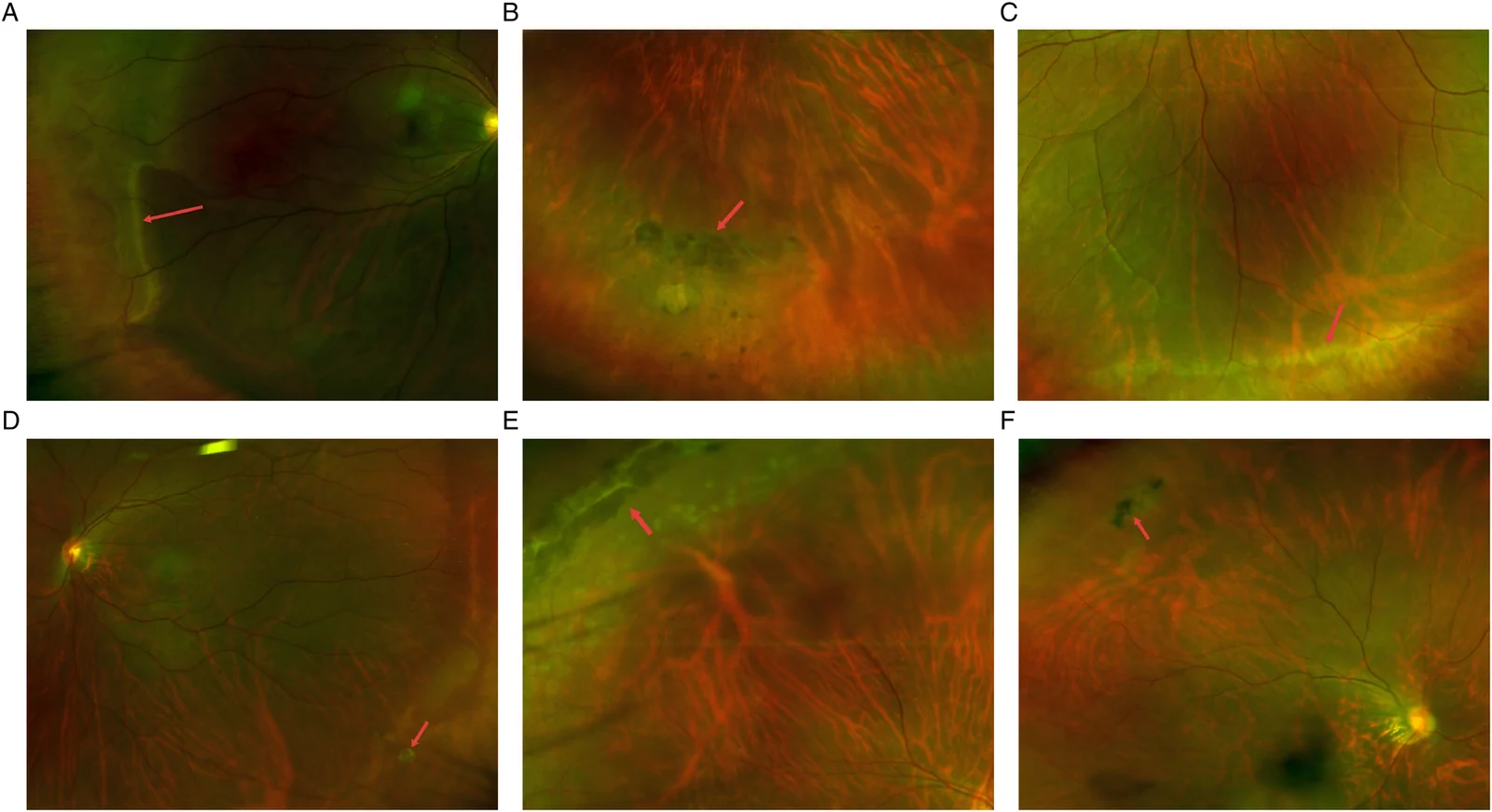
Representative ultra-widefield images of peripheral retinal lesions: **(A)** white without pressure; **(B)** lattice degeneration; **(C)** snowflake degeneration; **(D)** retinal tear; **(E)** snail-track degeneration; and **(F)** pigmentary degeneration.

**Table 4:** Proportion of different types of PRDs in the PRD group [eyes (%)].

White without pressure	Grid-like degeneration	Frost-like degeneration	Retinal tear	Cystoid degeneration	Snail-stack degeneration	Pigmentary degeneration
33 (47.14)	13 (18.57)	12 (17.14)	8 (11.43)	6 (8.57)	5 (7.14)	5 (7.14)

### 3.5 Quadrant distribution of PRLs

The 70 PRL eyes contained 82 lesions, of which 7 spanned two quadrants, yielding 89 quadrant involvements. These were distributed as follows: superior (24, 26.97%), superotemporal (22, 24.72%), inferior (18, 20.22%), inferotemporal (15, 16.85%), temporal (5, 5.62%), nasal (2, 2.25%), superonasal (2, 2.25%), and inferonasal (1, 1.12%). Thus, quadrant counts exceed lesion counts, and percentages use 89 quadrant involvements as the denominator. The distribution is shown in Figure 2.

**Figure 2:**
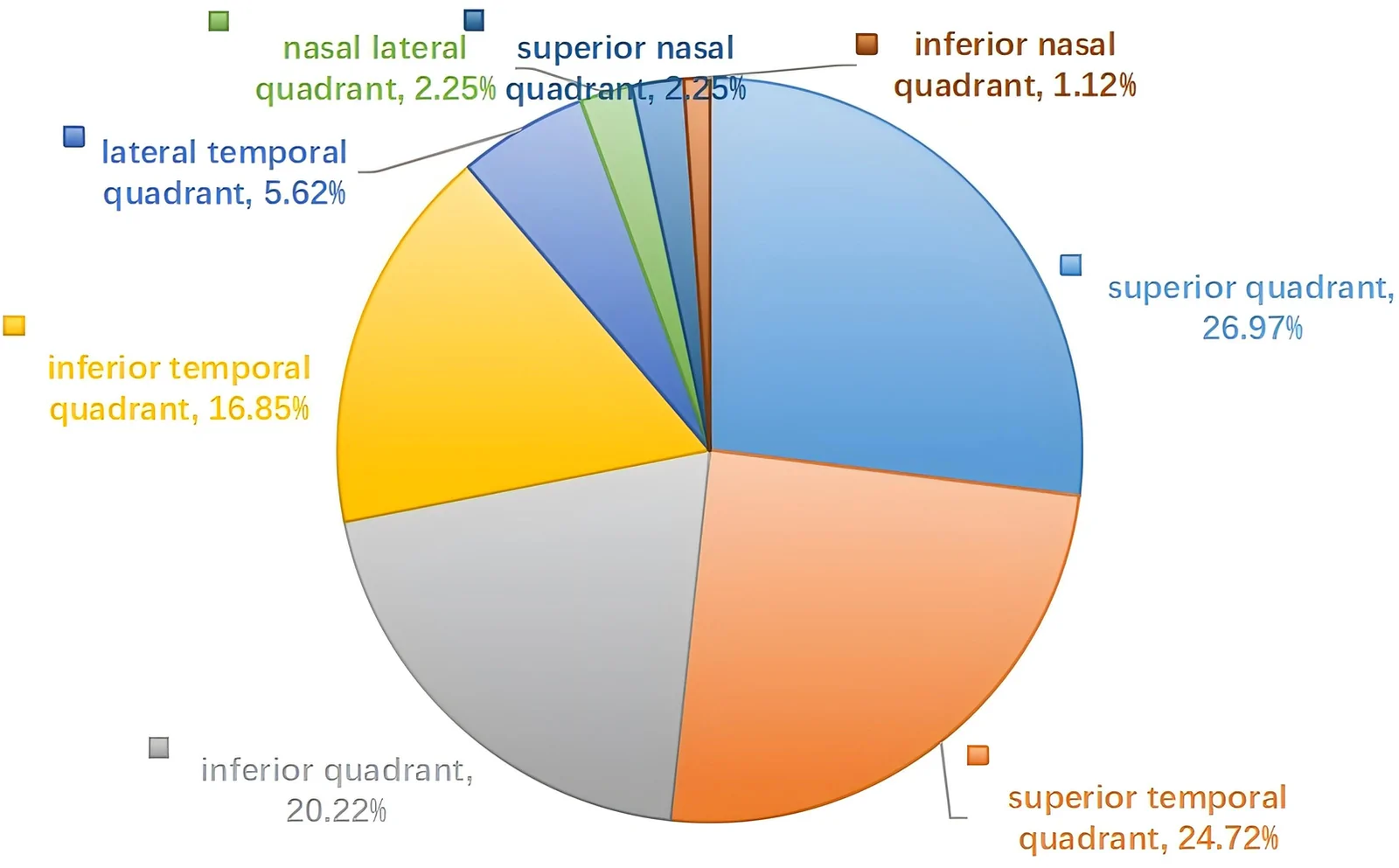
Distribution of peripheral retinal lesion involvement across eight retinal sectors in 70 eyes with peripheral retinal lesions (PRLs). The 82 lesions contributed 89 sector involvements because seven lesions extended across two adjacent sectors. Percentages were calculated using the 89 sector involvements as the denominator.

## 4 Discussion

As myopia becomes increasingly prevalent, attention has expanded from macular complications to peripheral retinal findings (36–38). Although many PRLs are asymptomatic, lesions accompanied by retinal holes, tears, or vitreoretinal traction may precede retinal detachment and irreversible visual impairment (39, 40). The present study describes PRLs in adults undergoing refractive-surgery evaluation and examines their unadjusted associations with ocular biometric parameters.

Participant age did not differ between the eye-defined groups, but the relatively restricted young-adult age distribution limited assessment of age-related effects. Previous studies have reported age- and sex-related differences in peripheral retinal findings and myopia (41–43). Participant-level sex data were not available in the analytic file; therefore, sex could not be evaluated as a potential confounder in the present analysis.

PRL eyes had a longer AL than NF eyes, and the unadjusted proportion of PRLs was higher among eyes with AL > 26.0 mm. These observations are consistent with previous reports linking longer AL with peripheral retinal changes in myopic eyes (41, 42, 44). Possible explanations include mechanical stretching of the retina and vitreous and degenerative retinal-choroidal alterations in elongated eyes (45, 46). Nevertheless, the cross-sectional design does not establish that AL elongation caused the observed lesions. SE did not differ between groups (47, 48), whereas Km and CR differed substantially^.^ SEAL was also more negative in PRL eyes. However, SEAL contains no AL term, and CR is mathematically related to Km (CR = 337.5/Km); it therefore cannot be interpreted as isolating the contribution of AL or as independent of corneal curvature. SEAL should be regarded as exploratory until its derivation, reproducibility, and clinical validity are independently evaluated.

White without pressure, lattice degeneration, and snowflake degeneration were the most frequently recorded lesions. The dominant quadrant involvements were superior, superotemporal, inferior, and inferotemporal. These findings are broadly consistent with reports that peripheral lesions in young myopic eyes commonly involve superior and temporal retinal regions (44). Because multiple lesions and multi-quadrant involvement occurred in some eyes, lesion-type and quadrant percentages are descriptive and should not be interpreted as mutually exclusive prevalence estimates.

This study has several limitations. First, it was a small, single-center study of patients attending a refractive surgery clinic, limiting generalizability to the broader population with mild-to-moderate myopia. Second, the analyses were cross-sectional and unadjusted; no multivariable logistic regression was performed, so independent associations and residual confounding by factors such as age and sex could not be assessed. Third, two retinal specialists independently assessed the fundus findings and disagreements were adjudicated, but interobserver agreement was not quantified and masking to biometric measurements was not documented. Fourth, SEAL is an unvalidated, study-specific transformation that is algebraically dependent on Km and CR and does not directly include AL. Finally, detailed peripheral OCT (49) or other structural imaging (50) was not available to investigate lesion mechanisms.

## 5 Conclusion

In adults with mild-to-moderate myopia attending a refractive surgery clinic, PRLs were associated at the eye level with longer AL, flatter corneas, and more negative exploratory SEAL values. These findings support careful peripheral retinal examination during preoperative assessment but do not establish independent predictors or causality. Larger, prospectively designed studies with validated lesion grading and adjusted analyses are required.

## Data Availability

The original contributions presented in the study are included in the article/Supplementary Material, further inquiries can be directed to the corresponding authors.
